# Association of Socioeconomic Status With Alpha Omega Alpha Honor Society Membership Among Medical Students

**DOI:** 10.1001/jamanetworkopen.2021.10730

**Published:** 2021-06-02

**Authors:** Mytien Nguyen, Hyacinth R. C. Mason, Patrick G. O’Connor, Marcella Nunez-Smith, William A. McDade, Darin Latimore, Dowin Boatright

**Affiliations:** 1 MD-PhD Program, Yale School of Medicine, New Haven, Connecticut; 2 Department of Medical Education and Family and Community Medicine, Albany Medical College, Albany, New York; 3 Section of General Internal Medicine, Yale School of Medicine, New Haven, Connecticut; 4 Accrediation Council for Graduate Medical Education, Chicago, Illinois; 5 Department of Emergency Medicine, Yale School of Medicine, New Haven, Connecticut

## Abstract

This cross-sectional study investigates the association between Alpha Omega Alpha (AΩA) honor society membership and medical student socioeconomic status.

## Introduction

Alpha Omega Alpha (AΩA) honor society membership is the hallmark of academic achievement in undergraduate medical education, and AΩA membership is associated with future success in academic medicine.^1^ AΩA members are chosen based on academic performance, leadership, patient care, and service.^2^ Nevertheless, studies have shown racial/ethnic disparities in AΩA membership.^3,4^ Whether similar disparities in AΩA membership exist across socioeconomic strata remains unknown. To address this knowledge gap, we investigated the association between AΩA membership and medical student socioeconomic status.

## Methods

In this cross-sectional study, we obtained deidentified data from the Association of American Medical Colleges (AAMC) describing medical students applying to residency between 2018 and 2020 who matriculated in the 2014-2015 and 2015-2016 academic years. We determined students’ AΩA membership through the AAMC’s data applications and services^5^ along with socioeconomic measures, including parental education, childhood household income, Pell grant assistance, and whether the student had been a beneficiary of state or federal financial assistance programs for low-income families. Additional characteristics included sex, self-reported race/ethnicity, and Medical College Admission Test (MCAT) scores. This study followed the Strengthening the Reporting of Observational Studies in Epidemiology (STROBE) reporting guideline and was approved by the Yale University institutional review board.

Our initial cohort included 36 617 students, from which we excluded 1729 (4.9%) because their medical school had no AΩA chapter, 3609 (9.8%) because AΩA elections occurred in senior year and results were not reported to the AAMC, and 1014 (2.7%) because the socioeconomic measures were unknown. Race/ethnicity was reported by students and categorized into the following groups: non-Hispanic White, non-Hispanic Asian, non-Hispanic Black or African American, Hispanic, non-Hispanic American Indian, Alaskan Native, Hawaiian Native, and other Pacific Islander, or other. Students who identified with more than 1 racial/ethnic category were categorized as multiracial.

We compared differences between AΩA members and nonmembers using the χ^2^ test. We used logistic regression to model the association between measures of socioeconomic status and AΩA membership, adjusting for students’ demographic characteristics and MCAT scores. Owing to the collinearity between childhood household income, Pell grant assistance, and state or federal financial aid, childhood household income was selected for inclusion in the multivariate model to provide the greatest distinction in economic advantage between students. Statistical analyses were performed using Stata, version 16.1 (StataCorp). A 2-sided *P* < .05 defined statistical significance.

## Results

Among 30 265 students in the study cohort, 4504 (14.9%) were first-generation college graduates, 9130 (30.2%) reported childhood household income less than $75 000, and 6862 (22.7%) and 5796 (19.2%) were Pell grant and state or federal financial assistance recipients, respectively. The percentage of students identifying as first-generation college graduates (598 of 5745 members [10.4%] vs 3906 of 24 519 nonmembers [15.9%]), reporting childhood household income less than $75 000 (1238 of 5745 members [21.4%] vs 7892 of 24 519 nonmembers [32.2%]), and receiving Pell grants (887 of 5745 members [15.4%] vs 5975 of 24 519 nonmembers [24.4%]) or state or federal financial assistance (746 of 5745 members [13.0%] vs 5050 of 24 519 nonmembers [20.6%]) was lower for AΩA members compared with nonmembers (*P* < .001) (Table 1).

**Table 1.** Characteristics of Medical Students by AΩA Honor Society Membership

Characteristic	AΩA Honor Society member, No. (%)		*P* value
	Yes (n = 5746 [19.0%])	No (n = 24 519 [81.0%])	
First-generation college graduate			
No	5148 (89.6)	20 613 (84.1)	<.001
Yes	598 (10.4)	3906 (15.9)	
Childhood household income, $^a^			
0-49 999	549 (9.6)	4541 (18.5)	<.001
50 000-74 999	689 (12.0)	3351 (13.7)	
75 000-124 999	1462 (25.4)	5813 (23.7)	
125 000-199 999	936 (16.3)	3040 (12.4)	
≥200 000	1005 (17.5)	2981 (12.1)	
I do not know or decline to answer^b^	1105 (19.2)	4793 (19.6)	
Pell grant recipient			
No	4859 (84.6)	18 544 (75.6)	<.001
Yes	887 (15.4)	5975 (24.4)	
State/federal financial assistance			
No	4596 (80.0)	17 199 (70.2)	<.001
Yes	746 (13.0)	5050 (20.6)	
I do not know or decline to answer^a^	404 (7.0)	2270 (9.2)	
Sex			
Male	2891 (50.3)	12 553 (51.2)	.23
Female	2855 (49.7)	11 966 (48.8)	
Race/ethnicity			
Non-Hispanic			<.001
White	3940 (68.6)	12 672 (51.7)	
Asian	806 (14.0)	4854 (19.8)	
Black or African American	111 (1.9)	1745 (7.1)	
American Indian, Alaskan Native, Hawaiian Native, or other Pacific Islander	14 (0.2)	85 (0.3)	
Multiracial	277 (4.8)	1452 (5.9)	
Hispanic	199 (3.6)	1588 (6.4)	
Unknown or other^c^	399 (6.9)	2123 (8.8)	
MCAT quartile			
First (lowest)	656 (11.4)	5751 (23.5)	<.001
Second	1514 (26.4)	7290 (29.7)	
Third	1380 (24.0)	4860 (19.8)	
Fourth (highest)	2196 (38.2)	6618 (27.0)	

Abbreviations: AΩA, Alpha Omega Alpha; MCAT, Medical College Admissions Test.

^a^ Household income level of family from birth to 18 years of age.

^b^ Students selected the option “I do not know” or “Decline to answer.”

^c^ Students’ race/ethnicity were unknown, or students selected other.

In our fully adjusted multivariable model, students reporting a childhood household income less than $125 000 (2700 of 5745 members [47.0%] vs 13 705 of 24 519 nonmembers [55.9%]) were less likely to be AΩA members than students reporting a household income of at least $200 000 (1005 of 5745 members [17.5%] vs 2981 of 24 519 nonmembers [12.1%]) (Table 2). This disparity was greatest for students reporting a childhood household income less than $50 000 (549 of 5745 members [9.6%] vs 4541 of 24 519 nonmembers [18.5%]), for whom the odds of AΩA membership were 46% lower than the odds for students whose childhood household income totaled $200 000 or more (adjusted odds ratio, 0.54; 95% CI, 0.48-0.62) (Table 2). Female students were more likely than male students to be members (adjusted odds ratio: 1.17; 95%CI: 1.10-1.24), and Asian (adjusted odds ratio, 0.50; 95% CI, 0.46-0.55), Black or African American (adjusted odds ratio, 0.33; 95% CI, 0.27-0.40), Hispanic (adjusted odds ratio, 0.61; 95% CI, 0.52-0.71), and multiracial students (adjusted odds ratio, 0.67; 95% CI, 0.58-0.76) were less likely to be AΩA members than White students. MCAT scores were strongly associated with AΩA membership (Table 2). We found no significant interaction between socioeconomic measures and race/ethnicity.

**Table 2.** Association Between AΩA Honor Society Membership and Medical Student Characteristics

Characteristic	Adjusted odds ratio (95% CI)		
	Model 1: socioeconomic measures	Model 2: socioeconomic measures, sex, and race/ethnicity	Model 3: fully adjusted
No.	30 265	30 265	30 265
First-generation college graduate			
No	1 [Reference]	1 [Reference]	1 [Reference]
Yes	0.87 (0.78-0.96)	0.89 (0.80-0.98)	0.97 (0.87-1.07)
Childhood household income, $			
≥200 000	1 [Reference]	1 [Reference]	1 [Reference]
0-49 999	0.38 (0.34-0.43)	0.49 (0.43-0.56)	0.54 (0.48-0.62)
50 000-74 999	0.63 (0.56-0.70)	0.70 (0.62-0.78)	0.74 (0.66-0.83)
75 000-124 999	0.75 (0.69-0.83)	0.80 (0.73-0.88)	0.82 (0.75-0.91)
125 000-199 999	0.91 (0.83-1.01)	0.94 (0.85-1.05)	0.95 (0.86-1.06)
I do not know or decline to answer^a^	0.69 (0.62-0.76)	0.74 (0.67-0.81)	0.74 (0.67-0.82)
Sex			
Male	NA	1 [Reference]	1 [Reference]
Female	NA	1.07 (1.01-1.13)	1.17 (1.10-1.24)
Race/ethnicity			
Non-Hispanic			
White	NA	1 [Reference]	1 [Reference]
Asian	NA	0.57 (0.52-0.61)	0.50 (0.46-0.55)
Black or African American	NA	0.24 (0.20-0.29)	0.33 (0.27-0.40)
American Indian, Alaskan Native, Hawaiian Native, or other Pacific Islander	NA	0.61 (0.35-1.08)	0.75 (0.42-1.33)
Multiracial	NA	0.64 (0.56-0.73)	0.67 (0.58-0.76)
Hispanic	NA	0.49 (0.42-0.57)	0.61 (0.52-0.71)
Unknown or other^b^	NA	0.65 (0.58-0.73)	0.61 (0.55-0.69)
MCAT quartile			
First (lowest)	NA	NA	1 [Reference]
Second	NA	NA	1.55 (1.40-1.72)
Third	NA	NA	2.14 (1.92-2.37)
Fourth (highest)	NA	NA	2.54 (2.30-2.81)

Abbreviations: AΩA, Alpha Omega Alpha; MCAT, Medical College Admissions Test; NA, not applicable.

^a^ Students selected the option “I do not know” or “Decline to answer.”

^b^ Students’ race/ethnicity were unknown, or students selected other.

## Discussion

Our study’s major finding is that students from backgrounds with lower income than their peers were less likely to be AΩA members. Consistent with prior studies,^2,3^ our results show persistent and striking racial/ethnic disparities in AΩA membership, even after adjusting for socioeconomic measures and standardized test scores. Our study has limitations. Childhood income was self-reported and may not fully reflect parental financial resources, including assets. Furthermore, our study involved a 2-year time frame and may not represent historical trends in AΩA membership. However, to our knowledge, this study is the largest and only examination of AΩA membership by socioeconomic status to date. Although AΩA membership has been recognized as a marker of merit, our findings suggest that AΩA membership may also be an indicator of privilege. Medical schools must ensure that the learning environment allows all students to thrive irrespective of their backgrounds.
